# Change of permanent grasslands extent (1996-2015) and national grassland dataset of Switzerland

**DOI:** 10.1016/j.dib.2018.09.039

**Published:** 2018-09-18

**Authors:** Simon Schmidt, Christine Alewell, Katrin Meusburger

**Affiliations:** a Environmental Geosciences, University of Basel, Bernoullistrasse 30, CH-4056 Basel, Switzerland; b Swiss Federal Institute for Forest, Snow and Landscape Research WSL, Zürcherstrasse 111, CH-8903 Birmensdorf, Switzerland

**Keywords:** Land use change, Land cover classification, Time series, Change detection, Soil erosion, Alpine environment, C-factor, CCI Land Cover

## Abstract

So far, neither a grassland map, temporal analysis of the conversion of permanent grassland (PG) to other land uses nor the differentiation of permanent and temporal grassland exists for Switzerland. For the first time in Switzerland, we present a Swiss national grassland map for the year 2015 capturing the extent of both, permanent and temporal grasslands (here called grasslands) by intersecting the information of three datasets. We blended the high temporal resolution Climate Change Initiate (CCI) Land Cover of 2015 (processed by the European Space Agency (ESA)), with the high spatial resolution Swiss topographical landscape model “SwissTLM3D” and the landscape model “vector25” both provided by Swisstopo. The final data presents the spatial patterns and the national extent of Swiss grasslands. Furthermore, the recently published (April 2017) CCI Land Cover dataset allow extracting the extent of grasslands for 24 years (1992–2015) with a coarse spatial resolution of 300 m. We used the time series data of the grassland extent to produce annual PG maps from 1996 to 2015. That data enables the identification of the development of grassland extent over two decades. The Swiss national grassland map is used for investigating the spatio-temporal patterns of the soil erosion risk of Swiss grasslands (see Mapping spatio-temporal dynamics of the cover and management factor (C-factor) for grasslands in Switzerland, doi:10.1016/j.rse.2018.04.008 (Schmidt el al., 2018)).

**Specifications table**

**Table:** 

Subject area	*Ecology*
More specific subject area	*Grassland mapping and land use change*
Type of data	*Figures (maps)*
How data was acquired	*Data were derived from Climate Change Initiative (CCI) Land Cover*[2], [3]*and Swisstopo*[4], [5]*. Data were processed for 2015 and an annual resolution for Switzerland for the years 1992/1996 to 2015*
Data format	*processed and analyzed data is available as Raster format (GeoTIFF) and Polygons (Shapefile)*
Experimental factors	*Details provided by the European Space Agency (ESA)*
Experimental features	*Grassland maps were extracted from the global CCI Land Cover*[2], [3]*and clipped for Switzerland. Two Swiss landscape models*[4], [5]*were used for the refinement of the grassland extent by clipping with additional topographical and land use information. Permanent grasslands and their change were derived by sets of five successive grassland maps.*
Data source location	*Switzerland*
Data accessibility	*The data are available with this article.*
Related research	[1] Schmidt, S., Alewell, C., Meusburger, K. (2018). Mapping spatio-temporal dynamics of the cover and management factor (C-factor) for grasslands in Switzerland. Remote Sensing of Environment, 211, 89–104. doi:10.1016/j.rse.2018.04.008.

**Value of the data**

• The data provide a first national map of the extent of Swiss grasslands which might not only be an important baseline data for ecological studies but also for multiple disciplines, e.g., alpine research, soil sciences, geosciences, agronomy, hydrology.
• Modelers and GIS-users are provided with a grassland map (2015) to distinct grasslands from other land use classes (e.g., arable land, forest).
• The separation of temporal and permanent grassland is feasible and of high relevance for ecological, geobotanical, biodiversity and soil research to interpret specific species composition and indicator for soil properties.
• The capturing of the conversion of permanent grassland from 1996 to 2015 is a valuable resource for future policy decision making.

## 1 Data

**Fig. 1:**
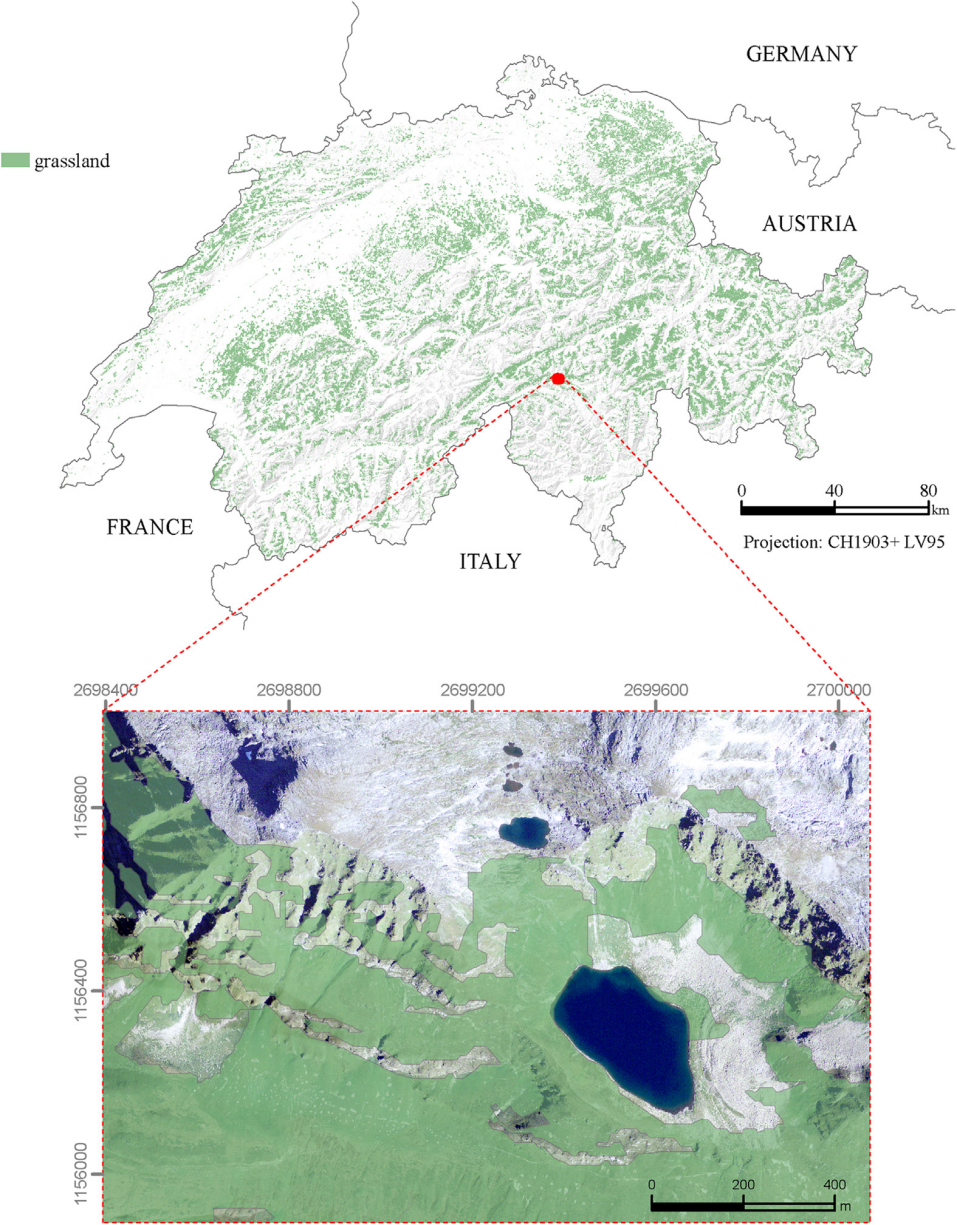
Refined Swiss national grassland map (spat. res. 300 m) of the year 2015. Temporal and permanent grassland is not distinguished here.

The presented map (Fig. 1) represents the extent of total grassland (no separation between temporal (TG) and permanent grasslands (PG)) for Switzerland for the year 2015. The comparison between the presented grassland map with digital orthophotos for 1000 random points reveals a mapping accuracy of grassland by 82.1%. The remaining of non-matching points (7.6%) is bedrock which is usually socialized with grassland. The remaining misclassified points correspond to 3.9% of forest areas, 2% of asphalted areas (e.g. streets), and 4.4% undefined land use types. The main cause for the mismatch is the coarse resolution of the grassland map pixels.

**Fig. 2:**
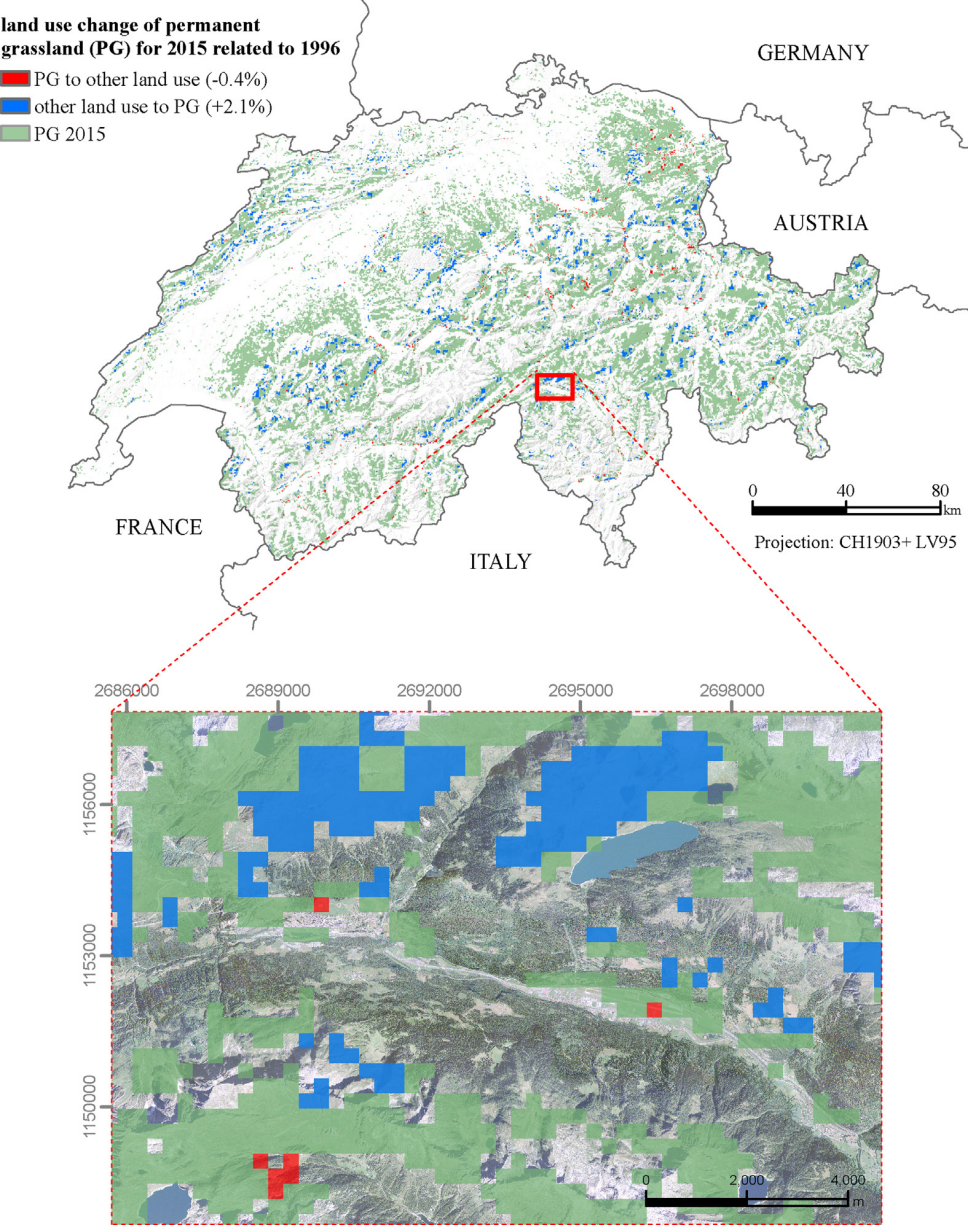
Land use change of permanent grassland in Switzerland for 2015 related to 1996 (spat. res. 300 m).

**Fig. 3:**
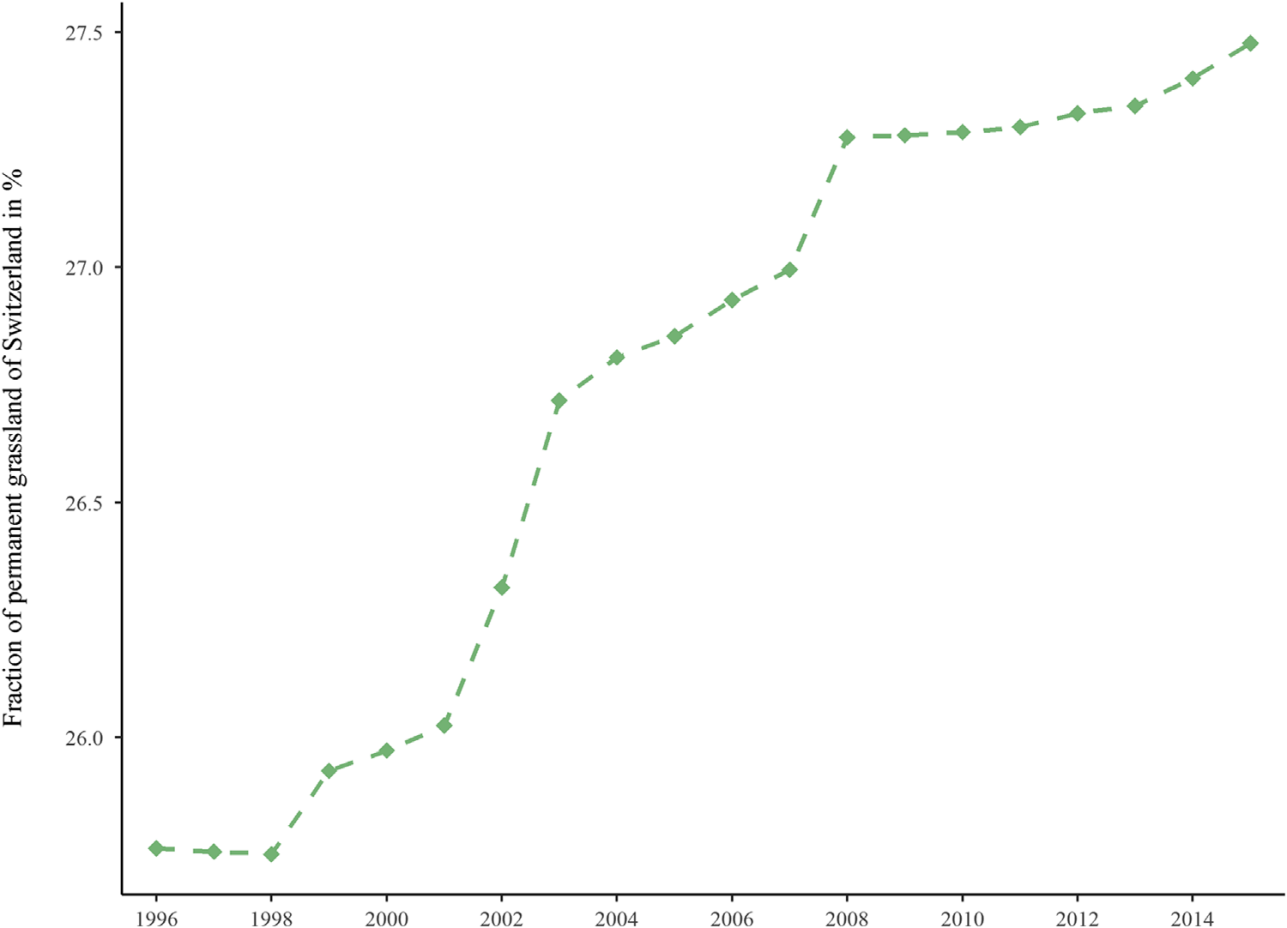
Fraction of permanent grassland from total area in Switzerland from 1996 to 2015 in percentages.

According to the Food and Agricultural Organization (FAO) definition, grassland is defined as “ground covered by vegetation dominated by grasses, with little or no tree cover” [6]. In contrast to TG, PG is not part of the crop rotation for a minimum of five successive years [8]. An overall gain (2.1%) of PG in 2015 compared to 1996 can be assessed (Fig. 2). About 0.4% of PG was converted to other land use units in the same comparative period. The PG time series over 20 years (1996–2015) shows a slight but continuously increasing trend from 1998 onwards (Fig. 3). The PG maps of the two decades are provided as enclosed data with this article. Soil properties vary with grassland type due to plowing and cultivation of TG. Therefore, the data, particularly when linked to agrarian development, planning, or soil degradation threats, are also a valuable resource for soil scientists. The Swiss national grassland map of 2015 (Fig. 1) was originally developed for investigating the spatio-temporal patterns of soil erosion risk on Swiss grasslands [1].

## 2 Experimental design, materials and methods

In 2017, the European Space Agency published annual globally available CCI Land Cover Maps (v2.0.7) including grassland for 24 consecutive years (1992–2015) with a spatial resolution of 300 m. We extracted the grasslands for all 24 years and clipped them to the Swiss national border [4]. The spatial resolution of 300 m represents a single class value of an area of 300 m × 300 m of the ground. Based on this data source we derived two grassland products: (i) the Swiss national grassland map for the year 2015 and (ii) the temporal change of permanent grassland areas in Switzerland from 1996 to 2015.

**Fig. 4:**
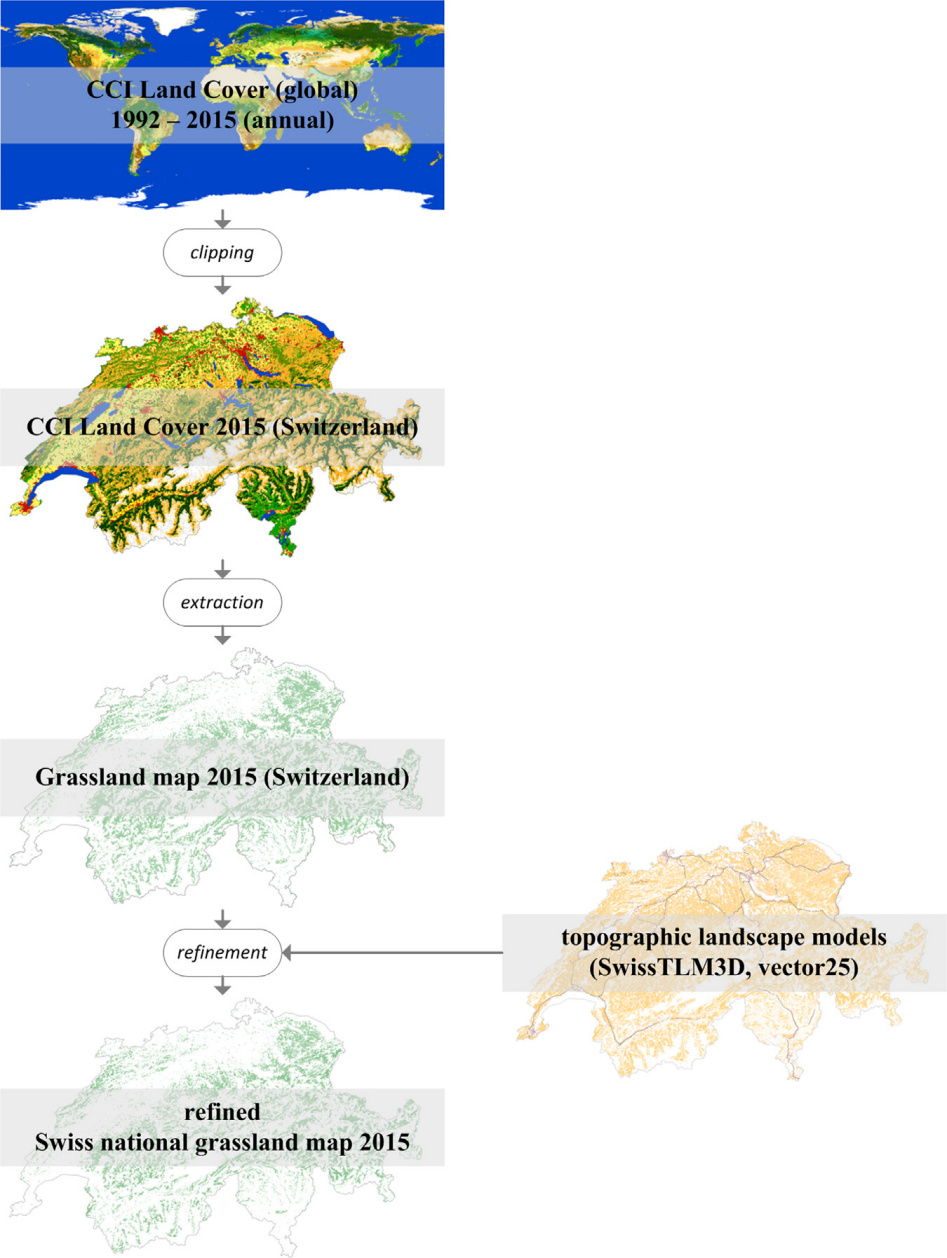
Flow chart for the processing of the refined Swiss national grassland map (2015).
  High spatial resolution digital orthophotos (0.25 m, SwissImage RGB, [7]) were used for validating the grassland map of Switzerland. A total of 1000 random points were set for a pseudo ground control within the here generated grassland map. These points are visual and statistical evaluated according to their real land use type.
(ii) The availability of grassland time series enables the extraction of PG from 1996 to 2015. Following the definition [8], we defined all grid cells as PG which represented grasslands in a succession of five years. PG maps could not be improved by clipping with the topographic landscape models (compare Fig. 1) owing to the lack of historical data of SwissTLM3D and vector25 [4], [5]. However, the investigation of the proportional change in PG is also feasible with the moderate-resolution of the CCI Land Cover grassland maps.

(i) We refined the extracted grassland class for the Swiss national grassland map of the year 2015 as they entail some generalization which affects primarily small landscape elements (e.g., streets, buildings) and other land use classes. For instance, small elements are not recorded as an individual class but assigned as grassland. The high resolution landscape models (geometric accuracy of 0.2–8 m; SwissTLM3D [4], vector25 [5]) of Switzerland increase the accuracy of the CCI Land Cover grassland map of 2015 by a clipping procedure due to its fine distinction of these landscape elements and land use classes. A flow chart of the processing is presented in Fig. 4. The landscape models contain a class (“Z_Uebrig”) which represents remaining primary areas such as grassland, arable land and so on which are not part of any other class and presented on a combined class level. That class is used for clipping to improve the accuracy of the CCI Land Cover maps of grassland. A grid cell remains grassland if a CCI Land Cover grassland grid cell matches with the Z_Uebrig polygon otherwise it is masked and a bad classification assumed due to the cell size. Furthermore, the buildings and streets (after buffering according to the mean street body width) were masked from the grassland map. Thereby, the accuracy of the map is increased, and misclassified landscape elements and land use classes are extracted.

## References

[1] Schmidt S., Alewell C., Meusburger K. (2018). Mapping spatio-temporal dynamics of the cover and management factor (C-factor) for grasslands in Switzerland. Remote Sens. Environ..

[2] O. Arino, F. Ramoino, Land cover CCI, product user guide version 2.0, Louvain-la-Neuve, 2017.

[3] S. Bontemps, M. Boettcher, C. Brockmann, G. Kirches, C. Lamarche, J. Radoux, M. Santoro, E. Vanbogaert, U. Wegmüller, M. Herold, F. Achard, F. Ramoino, O. Arino, P. Defourny, Multi-year global land cover mapping at 300 m and characterization for climate modelling, Achievements of the Land Cover component of the ESA Climate Change Initiative, ISPRS – International Archives of the Photogrammetry, Remote Sensing and Spatial Information Sciences XL-7/W3, 2015 323–328, 〈 10.5194/isprsarchives-XL-7-W3-323-2015〉.

[4] Swisstopo, SwissTLM3D, Wabern. 〈https://shop.swisstopo.admin.ch/en/products/landscape/tlm3D〉, 2017.

[5] Swisstopo, Vector25, Das digitale Landschaftsmodell der Schweiz, Wabern, 2007.

[6] J.M. Suttie, S.G. Reynolds, (Eds.), Grasslands of the world, Rome, 2005.

[7] Swisstopo, Swissimage, Das digitale Farborthophotomosaik der Schweiz, Wabern, 2010.

[8] Smit H.J., Metzger M.J., Ewert F. (2008). Spatial distribution of grassland productivity and land use in Europe. Agric. Syst..

