# Supplementary figures and images for: Change of permanent grasslands extent (1996-2015) and national grassland dataset of Switzerland
Source: Data Brief. 2018 Sep 18;20:1992–8. doi: 10.1016/j.dib.2018.09.039 (PMC6172429; doi:10.1016/j.dib.2018.09.039)

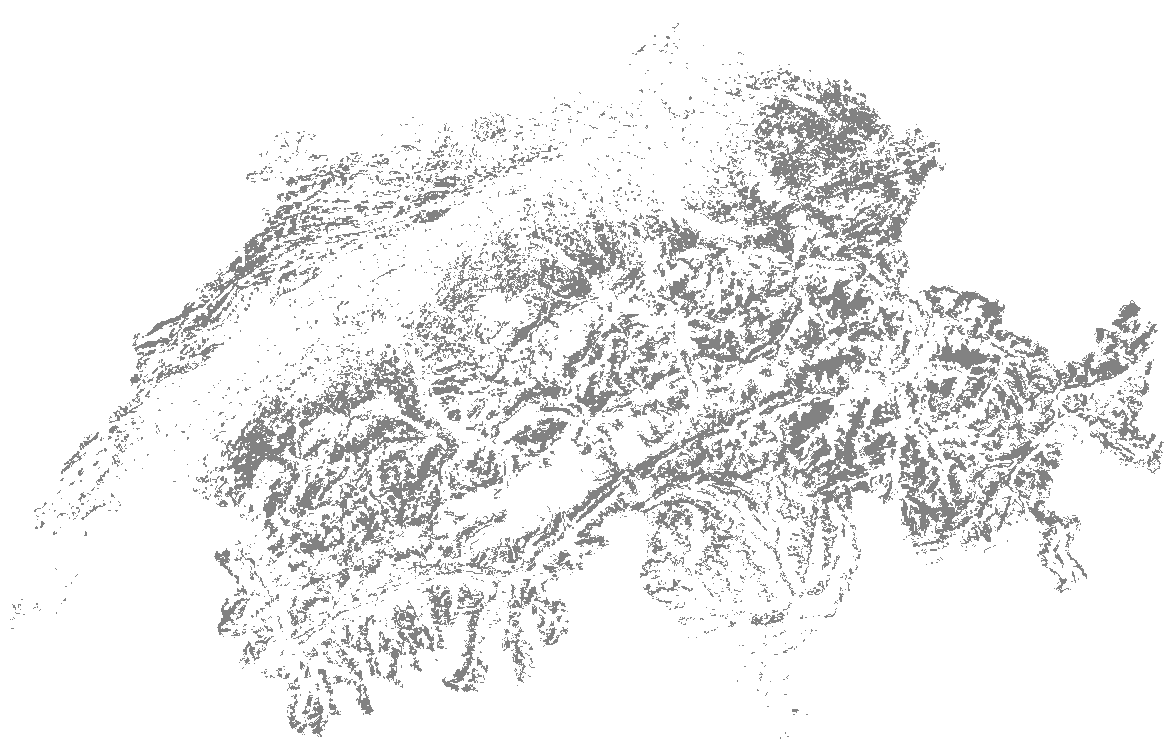

Supplement: Supplementary file 2 — Supplementary material [file mmc2.zip › Supplement Data/Swiss_national_CCILC_grassland_map_2015.tif]

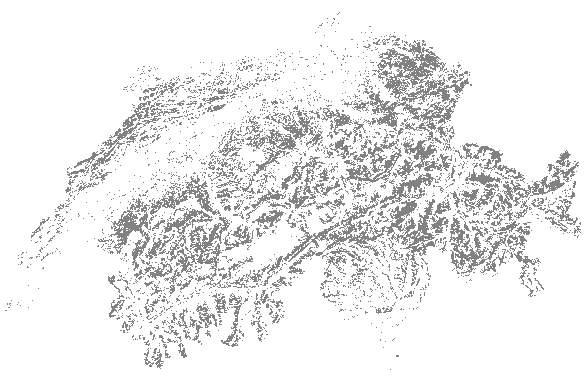

Supplement: Supplementary file 2 — Supplementary material [file mmc2.zip › Supplement Data/Swiss_national_CCILC_grassland_map_2015.tif.ovr]
